# HPV infection and bacterial microbiota in the semen from healthy men

**DOI:** 10.1186/s12879-021-06029-3

**Published:** 2021-04-21

**Authors:** Heidi Tuominen, Jaana Rautava, Katja Kero, Stina Syrjänen, Maria Carmen Collado, Samuli Rautava

**Affiliations:** 1 Department of Oral Pathology and Oral Radiology, Institute of Dentistry, Faculty of Medicine, University of Turku, Lemminkäisenkatu 2, FIN-20520 Turku, Finland; 2 Department of Oral and Maxillofacial Diseases, Clinicum, Faculty of Medicine, University of Helsinki and Helsinki University Hospital, Helsinki, Finland; 3 Department of Pathology, Medicum, Faculty of Medicine, University of Helsinki and HUS Diagnostic Center, HUSLAB, Helsinki University Hospital, Helsinki, Finland; 4 Department of Obstetrics and Gynaecology, University of Turku & Turku University Hospital, Turku, Finland; 5 Department of Pathology, Turku University Hospital, Turku, Finland; 6 Department of Biotechnology, Institute of Agrochemistry and Food Science, Spanish National Research Council (IATA-CSIC), Valencia, Spain; 7 Department of Paediatrics, University of Turku & Turku University Hospital, Turku, Finland; 8 Department of Pediatrics, University of Helsinki & Helsinki University Hospital, Helsinki, Finland

**Keywords:** Bacteria, HPV, Men, Microbiome, Microbiota, Semen

## Abstract

**Background:**

Aberrant microbiota composition has been linked to disease development at numerous anatomical sites. Microbiota changes in reaction to viral infections, such as human papillomavirus (HPV), have been investigated almost exclusively in the female reproductive tract. However, HPV infection may also affect male health by reducing semen quality and fertility. The aim of this study was to investigate whether present HPV DNA is associated with detectable changes in semen bacterial microbiota composition and diversity.

**Methods:**

This study relied on stored semen samples from 31 fertile healthy men who participated in the Finnish family HPV Study during the years 1998–2001. DNA was extracted from semen with PCR template preparation kit. HPV was genotyped using Luminex-based Multimetrix® assay. Microbiota was analyzed from the V3-V4 region of 16S rDNA gene following sequencing on an Illumina MiSeq platform. All statistical analyses were performed with Calypso software version 8.84.

**Results:**

HPV DNA was detected in 19.4% (6/31) of the semen samples. HPV status in the semen did not impact the α-diversity estimations, as measured by Chao1 and Shannon indices, nor ß-diversity. Nevertheless, HPV-positive semen samples exhibited differences in the taxonomic composition of the bacterial microbiota including higher abundances of *Moraxellaceae* (*p* = 0.028), *Streptococcus* (*p* = 0.0058) and *Peptostreptococcus* (*p* = 0.012) compared to HPV-negative semen samples.

**Conclusion:**

HPV infection is associated with altered bacterial microbiota composition in semen, and this might have in impact to male health in general. As of present, it is unclear whether these changes result from HPV infection or whether altered bacterial microbiota increases susceptibility to HPV infection. More research is needed on viral-bacterial interactions in the male reproductive system.

**Supplementary Information:**

The online version contains supplementary material available at 10.1186/s12879-021-06029-3.

## Background

Human papillomavirus (HPV) is a known oncovirus and it has potential to cause carcinoma in the genital, anal and oropharyngeal regions [1]. The natural history of HPV is currently well known in the female cervical region as well as in the oropharyngeal region. Nonetheless, HPV can infect other body sites as well [2, 3].

Several studies have reported the presence of HPV DNA in 2–31% of semen samples from healthy men [4–7]. Luttmer et al. [8] found that the presence of HPV in semen was associated with the presence of HPV in the penile scrapes but not with flat penile lesions (FPL). Foresta et al. [9] found HPV16 proteins in peripheral blood leukocytes in individuals with HPV-positive seminal samples. Therefore, suggesting the presence of HPV in semen seems not to be only local. HPV in semen has been implicated in male infertility. A systematic review including 21 original articles, concluded that HPV DNA found in semen is connected with reduced semen quality [10]. In a relatively recent study, women undergoing intrauterine insemination with HPV-positive semen were found to have four times fewer pregnancies than couples with HPV-negative semen [11]. To date, the association between the bacterial microbiota and HPV DNA in semen has yet remained unknown. Besides various factors, including sexual behavioural characteristics being determinant of the presence of HPV in semen particularly in adjusted analysis [8], other factors such as the semen microbiota could potentially impact the HPV status in semen. This speculation seems logical following the reports on concordance of HPV detection in semen and penile scrapes [8] and potential role of penile microbiota (specifically bacterial communities) on penile HPV infection [12]. Nonetheless, our knowledge about the interactions between HPV infection and the bacterial microbiota and its impact on human health is still limited.

Although limited, published literature in the last decade have given us a glimpse into the composition of semen microbiota. The semen microbiota is mainly composed of the genera *Lactobacillus*, *Pseudomonas*, *Prevotella*, *Gardenella*, *Corynebacterium, Staphylococcus* and *Streptococcus* [13–17]. Semen microbial composition has been associated with seminal health [13, 14, 16]. HIV infection, prostatitis (inflammation of the prostate gland) and sexual intercourse can all elicit an impact to the microbial composition in semen [12, 15, 16, 18]. According to previous studies, changes in the relative amount of different bacteria have been linked with semen quality [13, 14]. However, not all studies have been able to observe differences in semen bacterial microbiota between fertile and infertile couples [14, 17]. This could be explained by differences in study design, study populations or methods.

Since it is known that seminal microbiota can influence fertility and even offspring health, it is important to understand aspects that might affect semen microbiota composition [19]. We investigated the prevalence in HPV positivity in a subset of samples from Finnish Family HPV Study and evaluated the association between HPV and bacterial microbiota in these semen samples.

## Methods

### Study design, study population and ethical consideration and consent

The subjects and samples of the present study were from a sub-set of subjects from the Finnish Family HPV Study collected during the years 1998–2001 [20, 21]. The original study was designed to evaluate the interactions of HPV infection within families. Written informed consent was obtained from all participants. The study design was found acceptable by the Ethics Committee of the Intermunicipal Hospital District of Southwest Finland (#3/1998, #2/2006, 45/180/2010).

**Fig. 1:**
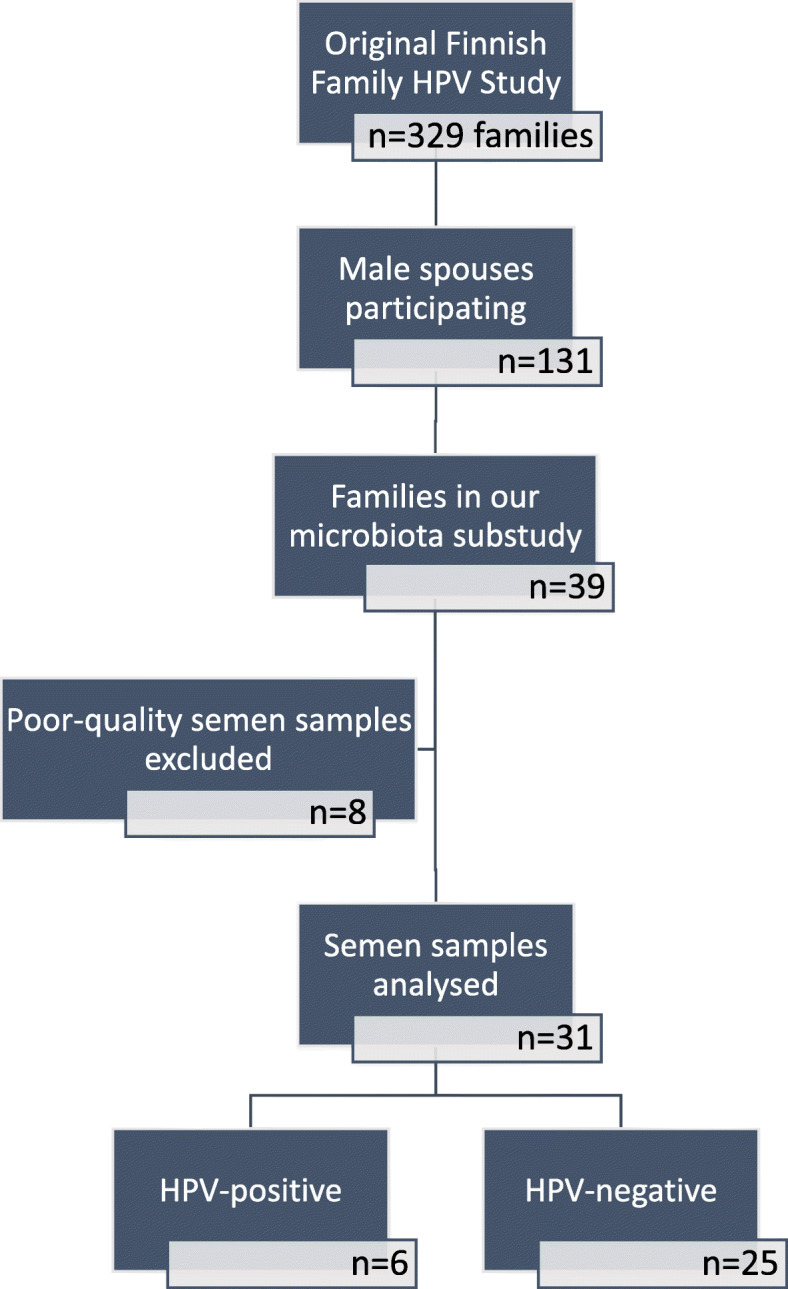
A flow chart depicting sample selection from the original Finnish Family HPV study

Semen samples for microbiota analyses were collected based on the placenta HPV status as previously described in detail [3]. The original inclusion criteria included 13 mothers wih HPV-positive placenta samples and 26 mothers with HPV-negative placenta samples (13 from caesarean section deliveries and another 13 from vaginal deliveries) in addition with male spouse and infant samples. Study subjects were excluded if some of the sample type was missing. Altogether, 31 semen samples from the male spouses were available for the present study. The sample selection and detailed information about the samples have been described in detail in Fig. 1**,** Table 1 and Supplementary Table 1.

**Table 1:** The list of the samples

ID	Male spouse’s age-range (years)	Smoking	Semen (HPV type)	Female spouse’s HPV status (genotype)
1	20–24	yes	–	oral (16)
2	20–24	no	–	–
3	30–34	yes	–	–
4	25–29	no	–	–
5	20–24	no	18	oral (16)
6	20–24	yes	–	oral (16)
				cervix (16,33)
7	20–24	yes	–	–
8	30–34	yes	6	–
9	25–29	no	6, 33	–
10	25–29	no	–	–
11	20–24	no	–	–
12	25–29	no	–	–
13	40–44	no	–	–
14	30–34	no	33	oral (16)
15	35–39	no	–	–
16	30–34	no	–	placenta (6)
17	25–29	yes	–	cervix (16,31,66)
18	20–24	no	–	–
19	30–34	no	–	–
20	25–29	no	–	placenta (6)
				cervix (6,59)
21	25–29	yes	–	–
22	35–39	no	16	–
23	25–29	no	–	oral (16)
				cervix (16)
				placenta (16)
24	n.a.	n.a.	–	
25	25–29	no	–	
26	30–34	yes	–	oral (6)
				cervix (6)
				placenta (6)
27	20–25	yes	–	oral (58)
28	25–29	yes	–	–
29	30–34	no	53	oral (16)
30	20–25	no	–	oral (6,16,42,56)
				placenta (6)
31	25–29	no	–	oral (16)

Men’s age-ranges, smoking status and semen HPV status and genotype have been listed in addition with female spouse’s HPV status of three anatomical regions (placenta, cervix, oral). The female genital and oral samples have been obtained during the 3rd trimester of pregnancy

*n.a.* not available

Semen samples were collected during the female spouse’s third trimester. The detailed sample collection is described earlier [4]. Semen sample was self-collected into a sterile plastic container from each of the participants after 2–10 days of abstinence (mean: 4.4 days). The sample was transferred to the laboratory and placed in 37 °C for 30 min for liquefaction. The semen samples were centrifuged at 3500 rpm for 15 min and cellular fraction (pellet) and seminal plasma (secretions without semen cells e.g. supernatant) were stored. The fractions were stored separately first at − 20 °C, and subsequently kept in − 70 °C. Only the cellular fraction was used for the present study and for HPV DNA detection.

### HPV detection and genotype

Detailed description about the semen HPV DNA detection and genotyping is described earlier [7]. HPV DNA was extracted from frozen semen cellular fractions with the high purity polymerase chain reaction (PCR) template preparation kit (Roche Diagnostics, Switzerland), according to the manufacturer’s instructions. For HPV testing, nested PCR was performed using MY09/MY11 as external primers and GP05+/bioGP06+ as internal primers, because viral load/cell and number of infected cells among the uninfected cells was expected to be low. HPV genotyping was performed using the Luminex-based Multimetrix® assay (Progen Biotechnik GmbH, Heidelberg, Germany), which detects 24 HPV types; 6 low-risk genotypes (HPV6, 11, 42, 43, 44, and 70); 3 putative high-risk genotypes (HPV26, 53 and 66; and 15 high-risk genotypes (HPV16, 18, 31, 33, 35, 39, 45, 51, 52, 56, 58, 59, 68, 73 and 82). The assay was performed according to manufacturer’s instructions except only half of the volume of the recommended volume was used. In the final step 100 ul of the blocking buffer was used to measure the hybridized beads with Luminex LX-100 analyzer. The HPV genotypes of all of the male samples has been analyzed and reported earlier [7].

### 16S rRNA gene sequencing and analysis

Sample analyzing methods have previously been described in detail [2, 3, 22]. Briefly, the V3-V4 region of 16S rDNA gene was amplified following Illumina protocols using Nextera XT Index Kit (Illumina, San Diego, CA, USA). Libraries were sequenced on a MiSeq Illumina platform (Lifesequencing sequencing service, Valencia, Spain) and PCR amplification and library controls were sequenced along as negative controls to rule out and control any possible contaminations. Quality assessment of the raw sequence files was performed using FASTX-toolkit version 0.0.13 as described earlier [3]. An open-reference operational taxonomic unit (OTU) picking method using 99% identity to the Greengenes 13_8 database [23] was performed using QIIME pipeline (version 1.9.0) [24]. Calypso software version 8.84 (http://cgenome.net/calypso/) [25] was used with total sum normalization (TSS) combined with square root transformation for the differential analysis and diversity estimations. We considered *p*-values ≤0.05 statistically significant.

## Results

### HPV detection in semen samples

HPV DNA was detected in 6 of the 31 semen samples (19.4%). Infection with a single HPV genotype was found in five of the samples (HPV 16, 6, 18, 33 and 53; 3.2% for each), as seen in Table 1. Infection with multiple HPV types (HPV 6 and 33) was found in one case (3.2%).

None of the HPV DNA positive semen samples associated with female spouse’s HPV status in oral, cervix or from the placenta as we did not observe the same HPV genotype in partners simultaneously.

### Microbiota and HPV in semen

The predominant bacterial families detected in the semen samples consisted of *Comamonadaceae* (relative abundance 4.13%), *Bifidobacteriaceae* (2.44%), *Tissirellaceae* (2.39%) and *Corynebacteriaceae* (2.11%). In addition, *Delftia* (3.76%), *Unclassified Comamonadeceae* (1.56%), *Propionibacterium* (1.55%) and *Streptococcus* (1.52%) genera were detected. The most prevalent bacteria detected from HPV-negative and HPV-positive semen samples are presented in Supplementary Table 2.

**Fig. 2:**
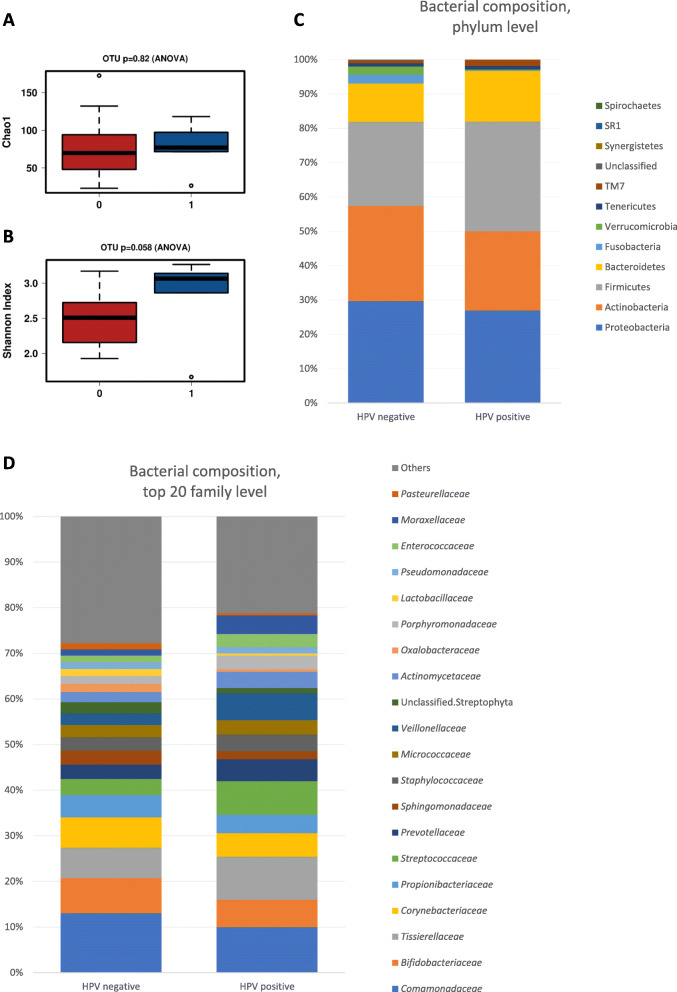
The bacterial microbiota in semen samples positive and negative for HPV**.** No difference was observed in diversity as measured by Chao1 index (**a**) and Shannon index (**b**) between HPV-positive (blue) and HPV-negative (red) semen samples. The relative abundances of bacteria are presented on the phylum (**c**) and family (**d**) levels

HPV status in semen did not impact the alpha diversity estimations, as measured by two alpha diversity metrics: Chao1 (HPV-negative: 65 versus HPV-positive 75, *p* = 0.820, Fig. 2a) and Shannon index (HPV-negative: 2.5 versus HPV-positive 3.3, *p* = 0.058, Fig. 2b), although there was a trend towards increased diversity with the latter metric. No differences were detected in beta diversity observed with principal coordinates analysis (PCoA) with Bray-Curtis distance (ADONIS *p* = 0.494) or redundancy analysis (RDA, *p* = 0.506) as presented in the Supplementary Data 1.

Since there was no major impact to semen bacterial microbiota composition in general according to HPV status, we sought to examine if there were any differentially abundant taxa in HPV-negative and HPV-positive semen samples. Indeed, semen HPV status was associated with differences in the relative abundance of specific bacterial taxa. At phylum level, Fusobacteria were more abundant in HPV-negative (relative abundance: 0.450%) compared to HPV-positive (0.032%) samples, *p* = 0.038 (Fig. 2c). At family level, *Streptococcaceae* (relative abundance: 2.60% versus 1.27%, *p* = 0.006), *Peptostreptococcaceae* (0.49% versus 0.063%*, p* = 0.016), *Veillonellaceae* (2.10% versus 0.80%, *p* = 0.026) *and Moraxellaceae* (1.45% versus 0.49%, *p* = 0.028) were more abundant in the HPV-positive samples than in HPV-negative samples (Fig. 2d). HPV-positive samples presented higher relative abundance of the genera *Streptococcus* (2.60% versus 1.27%, *p* = 0.006), *Serratia* (0.18% versus 0.01%, *p* = 0.008), *Dialister* (1.50% versus 0.42%, *p* = 0.009) and *Peptostreptococcus* (0.45% versus 0.039%, *p* = 0.012) compared to HPV-negative semen samples (Fig. 2e). At OTU level, higher levels of *Streptococcus oralis* (0.017% versus 0.00%, *p* = 0.002), *Anaerococcus mediterraneensis* (1.04% versus 0.12%, *p* = 0.003) and *Streptococcus anginosus* (0.025% versus 0.0016%, *p* = 0.011) were observed in the HPV-positive semen group relative to the HPV-negative group.

## Discussion

In the present study, we detected that the presence of HPV in semen is associated with some detectable changes in the semen bacterial microbiota composition in healthy individuals. The bacterial microbiota of the semen samples consisted mainly of *Comamonadaceae*, *Bifidobacteriaceae, Tissirellaceae, Corynebacteriaceae*, *Delftia*, unclassified *Comamonadeceae*, *Propionibacterium* and *Streptococcus* members. The overall seminal bacterial composition has been seen to vary between different studies [13, 14]. Previous studies have reported that the genera *Lactobacillus*, *Corynebacterium*, *Streptococcus*, *Pseudomonas*, *Prevotella* and *Gardenella* mainly constitute the healthy/normal semen bacterial microbiota [13, 14, 17]*.* The bacterial composition in our semen samples were detected to be somewhat different compared to previous studies, but overall, the genera *Corynebacterium* and *Streptococcus* were consistent. The small sample size may have an effect on our results.

The semen microbiota can be influenced by the bacteria colonized the penis, urethra and from the urine [12, 19, 26–30]. Contamination from skin bacteria from the participants’ hands is also possible. The circumcision status of the men is seen to change the penile microbiota composition as well [26, 28]. The Finnish Family HPV Study did not collect information about the male circumcision status, but in Finland it is not customary.

In the HPV-negative samples we detected higher abundance of Fusobacteria when compared to HPV-positive semen. Fusobacteria is a known oral pathogen [31] which has not, to authors’ best knowledge, been detected in human semen samples before. In the female reproductive health, on the other hand, Fusobacteria have been detected to play a part in cervicovaginal dysbiosis and HPV persistence [32].

Given that semen HPV and microbiota has been implicated in seminal health and disease/disorders, including infertility [10, 11, 13, 19, 33] and that the impact of HPV infection on semen microbiota remains unknown, we characterized the semen microbiota in presumably fertile men with and without HPV infection. All men in our study were presumably fertile, since they were recruited in the Finnish Family HPV Study as fathers-to-be. In our study cohort, the prevalence of HPV positivity in semen samples was 19.4%. This was within the range (2 to 31%) that has been reported in previous studies [4–6, 34, 35]. The prevalence of HPV has been reported to be slightly higher among men affected by unexplained infertility [5, 10, 36]. Furthermore, HPV infection has been associated with negative changes in semen quality including impairment of sperm motility and presence of anti-sperm antibodies [5, 37]. However, in our study, semen analysis (including sperm count test) was not performed to ascertain male fertility.

In our study, we detected HPV-positive semen samples to be more abundant with *Streptococcaceae, Peptostreptococcaceae*, *Veillonellaceae*, in addition with *Streptococcus, Serratia, Dialister and Peptostreptococcus* (Supplementary Table 1). In line with our findings, present HR-HPV (high-risk HPV) infection has been connected with e.g. *Prevotella* and *Dialister* in one recent study [12]. Furthermore, asymptomatic STI (sexually transmitted infections) have been shown to be more frequent in male with specific bacterial urine microbiota (mainly with *Prevotella* and *Veillonella*) [29].

Previous studies have connected *Lactobacillus, Streptococcus, Veillonella* and *Peptostreptococcus* with undefined male infertility [38, 39]. While HPV DNA found in semen has been linked to reduced semen quality*,* the abundances of *Lactobacillus* and *Streptococcus* have been identified to be significantly higher in semen samples from healthy men [13, 14]. In contrast to the present study, HPV DNA positive semen samples were associated with an increase of e.g. *Streptococcus* and *Peptostreptococcus* genera as compared to HPV DNA negative semen. Among the bacterial microbiota found in semen, *Ureaplasma* has been described as a known pathogen connected strongly with male infertility [40]. Both HPV infection and certain bacterial species, such as *Anaerococcus* in semen have been associated in inferior semen quality in previous studies [5, 10, 14, 36]. Male infertility is a complex multifactorial issue and cannot be explained by a single factor, such as HPV or presence of certain bacterial species. Furthermore, the connection between semen bacterial microbiota composition to male infertility is anything but simple [17]. Nevertheless, it is therefore important to understand how HPV and bacterial microbiota interact and what impact this interaction might have on male reproductive health.

Previously, it has been shown that present viral infection with HIV can shape the bacterial microbiota composition and that the altered bacterial composition might enable viral transmission in addition with changes in the pro-inflammatory cytokines [15]. It could be speculated that HPV might be able to interact with the bacterial microbiota in similar manner [33].

This study has some limitations. Our study design restricts us from making definitive conclusions about the viral-bacterial associations. Furthermore, the relatively low number of samples in general and the low number of HPV-positive semen samples may have an impact on the results, as we did not find significant impact of seminal HPV on the diversity of semen microbiota. HPV DNA may also originate from the proximal excurrent ducts (including efferent ductules, the vas epididymis and the proximal vas deferens) and not from HPV-infected seminal cells [8, 41]. The small number of samples is a limitation of the study and does not allow generalized conclusions Furthermore, the time of abstinence is based only on the participant’s own reports and might affect the sample quality and bacterial composition [18]. In addition, only the cellular fraction of the semen was used for DNA extraction and we cannot completely rule out that there could not be small traces of bacteria present in the seminal plasma. Nevertheless, this is the first study to show that seminal HPV infection is associated with high relative abundances of specific bacteria in the semen microbiota of presumably healthy men.

## Conclusions

In this study, we showed that men with seminal HPV DNA had higher relative abundance of specific seminal bacteria compared to men without seminal HPV DNA, but the causal relationship remains to be determined in future studies. Furthermore, data is largely lacking on whether the interplay between seminal HPV and semen microbiota has any clinical significance in male reproductive health.

Improvement of semen quality and securing healthy semen microbiota may be among the beneficial effects of HPV vaccination. Recently, HPV vaccination has been shown to improve the clearing of semen HPV infections and the reproductive outcomes in infertile patients with semen HPV infection [42].

## Supplementary Information

**Additional file 1:** **Table 1.** The list of the samples.

**Additional file 2:** **Supplementary Table 2.** A) The most prevalent bacteria detected in HPV-negative semen samples. B) The most prevalent bacteria detected in HPV-positive semen samples.

**Additional file 3:** **Supplementary Data 1.** The beta-diversity estimations (0 = HPV-negative semen samples, 1 = HPV-positive semen samples).

## Data Availability

The datasets used and/or analyzed during the current study are available from the corresponding author on reasonable request.

## Abbreviations

FPL: Flat penile lesions
HPV: Human papillomavirus
HR-HPV: High-risk human papillomavirus
OUT: Operational taxonomic unit
PCoA: Principal coordinates analysis
PCR: Polymerase chain reaction
RDA: Redundancy analysis
STI: Sexually transmitted infections
TSS: Total sum normalization
