# Supplementary material for: HPV infection and bacterial microbiota in the semen from healthy men
Source: BMC Infect Dis. 2021 Apr 21;21:373. doi: 10.1186/s12879-021-06029-3 (PMC8059035; doi:10.1186/s12879-021-06029-3)
Supplement: Supplementary file 1 — Additional file 1: Table 1. The list of the samples. [file 12879_2021_6029_MOESM1_ESM.docx]

|  | HPV-positive | HPV-negative |
| --- | --- | --- |
| Age range | 20-34 | 20-44 |
| Current smoking | 17% (1/6) | 35% (9/26) |
| Female spouse HPV-positive | 50% (3/6) | 35% (9/26) |
| Total | 19% (6/31) | 84% (26/31) |

**Supplementary Table 1: The list of the samples.**
