# Supplementary material for: HPV infection and bacterial microbiota in the semen from healthy men
Source: BMC Infect Dis. 2021 Apr 21;21:373. doi: 10.1186/s12879-021-06029-3 (PMC8059035; doi:10.1186/s12879-021-06029-3)
Supplement: Supplementary file 2 — Additional file 2: Supplementary Table 2. A) The most prevalent bacteria detected in HPV-negative semen samples. B) The most prevalent bacteria detected in HPV-positive semen samples. [file 12879_2021_6029_MOESM2_ESM.docx]

**Supplementary Table 2.**

1. **The most prevalent bacteria detected in HPV-negative semen samples.**

| **Phylum** | **Class** | **Order** | ***Family*** | ***Genus*** | ***Prevalence*** |
| --- | --- | --- | --- | --- | --- |
| Proteobacteria | Betaproteobacteria | Burkholderiales | *Comamonadaceae* | *Delftia* | *3.89%* |
| Actinobacteria | Actinobacteria | Corynebacteriales | *Corynebacteriaceae* | *Corynebacterium* | 2.17% |
| Actinobacteria | Actinobacteria | Bifidobacteriales | *Bifidobacteriaceae* | *Bifidobacterium* | 1.82% |
| Proteobacteria | Betaproteobacteria | Burkholderiales | *Comamonadaceae* |  | 1.64% |
| Actinobacteria | Actinobacteria | Propionibacteriales | *Propionibacteriaceae* | *Propionibacterium* | 1.59% |
| Firmicutes | Bacilli | Lactobacillales | *Streptococcaceae* | *Streptococcus* | 1.27% |
| Firmicutes | Clostridia | Clostridiales | *Peptoniphilaceae* | *Finegoldia* | 1.16% |
| Bacteroidetes | Bacteroidia | Bacteroidales | *Prevotellaceae* | *Prevotella* | 1.03% |
| Firmicutes | Clostridia | Clostridiales | *Clostridiales incertae sedis xi* | *Anaerococcus* | 1.01% |
| Firmicutes | Bacilli | Bacillales | *Staphylococcaceae* | *Staphylococcus* | 0.97% |

1. **The most prevalent bacteria detected in HPV-positive semen samples.**

| **Phylum** | **Class** | **Order** | ***Family*** | ***Genus*** | ***Prevalence*** |
| --- | --- | --- | --- | --- | --- |
| Proteobacteria | Betaproteobacteria | Burkholderiales | *Comamonadaceae* | *Delftia* | 3.25% |
| Firmicutes | Bacilli | Lactobacillales | *Streptococcaceae* | *Streptococcus* | 2.6% |
| Firmicutes | Clostridia | Clostridiales | *Clostridiales incertae sedis xi* | *Anaerococcus* | 2.04% |
| Actinobacteria | Actinobacteria | Corynebacteriales | *Corynebacteriaceae* | *Corynebacterium* | 1.83% |
| Bacteroidetes | Bacteroidia | Bacteroidales | *Prevotellaceae* | *Prevotella* | 1.72% |
| Firmicutes | Clostridia | Clostridiales | *Peptoniphilaceae* | *Peptoniphilus* | 1.62% |
| Firmicutes | Negativicutes | Selenomonadales | *Veillonellaceae* | *Dialister* | 1.50% |
| Firmicutes | Clostridia | Clostridiales | *Peptoniphilaceae* | *Finegoldia* | 1.48% |
| Actinobacteria | Actinobacteria | Bifidobacteriales | *Bifidobacteriaceae* | *Bifidobacterium* | *1.40%* |
| Actinobacteria | Actinobacteria | Propionibacteriales | *Propionibacteriaceae* | *Propionibacterium* | 1.38% |
