# Supplementary material for: HPV infection and bacterial microbiota in the semen from healthy men
Source: BMC Infect Dis. 2021 Apr 21;21:373. doi: 10.1186/s12879-021-06029-3 (PMC8059035; doi:10.1186/s12879-021-06029-3)

**Supplementary Data 1.**

**The beta-diversity estimations** (0=HPV-negative semen samples, 1=HPV-positive semen samples)

1. **PCoA (principal coordinates analysis)**


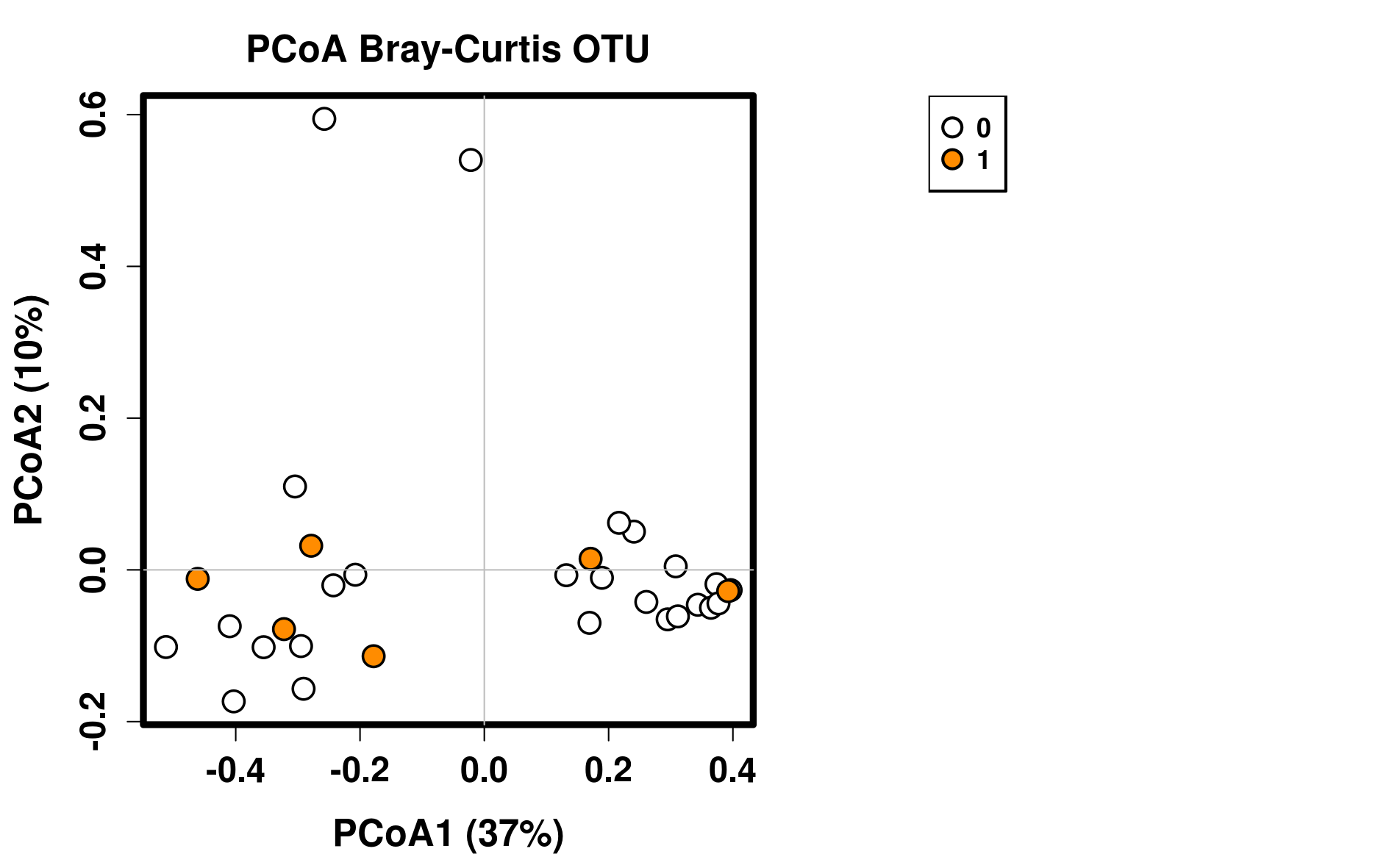


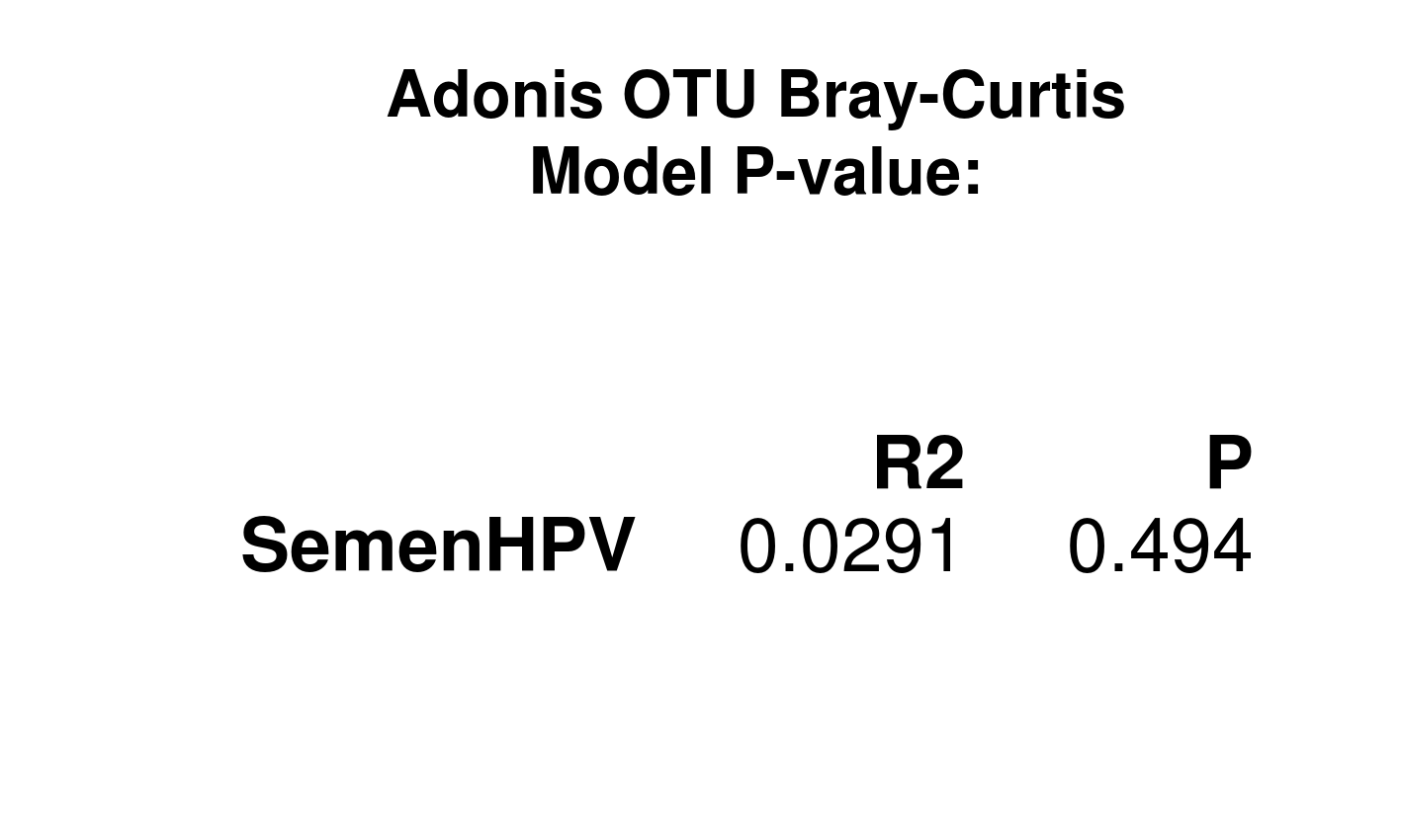


1. **RDA (redundancy analysis)**


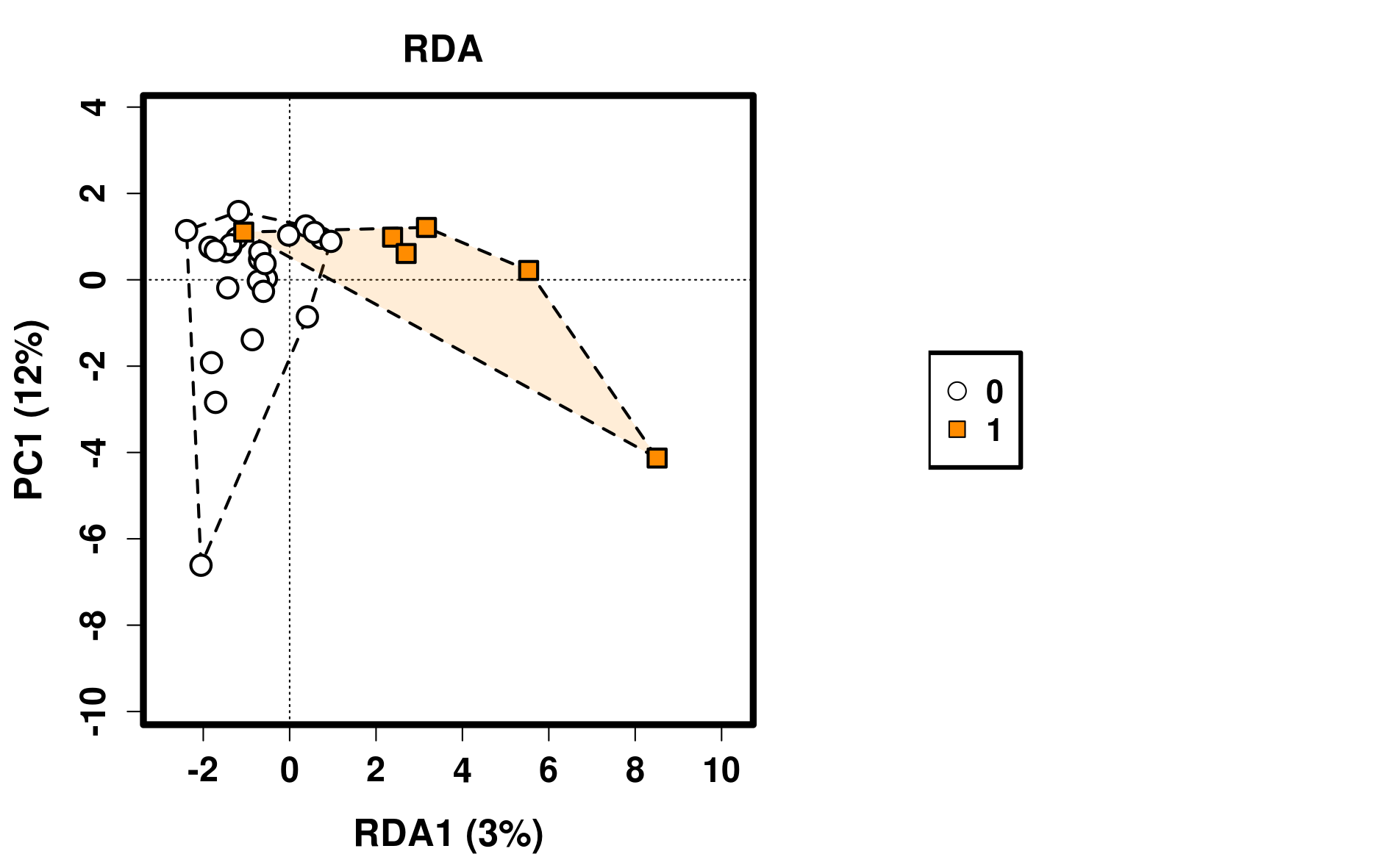


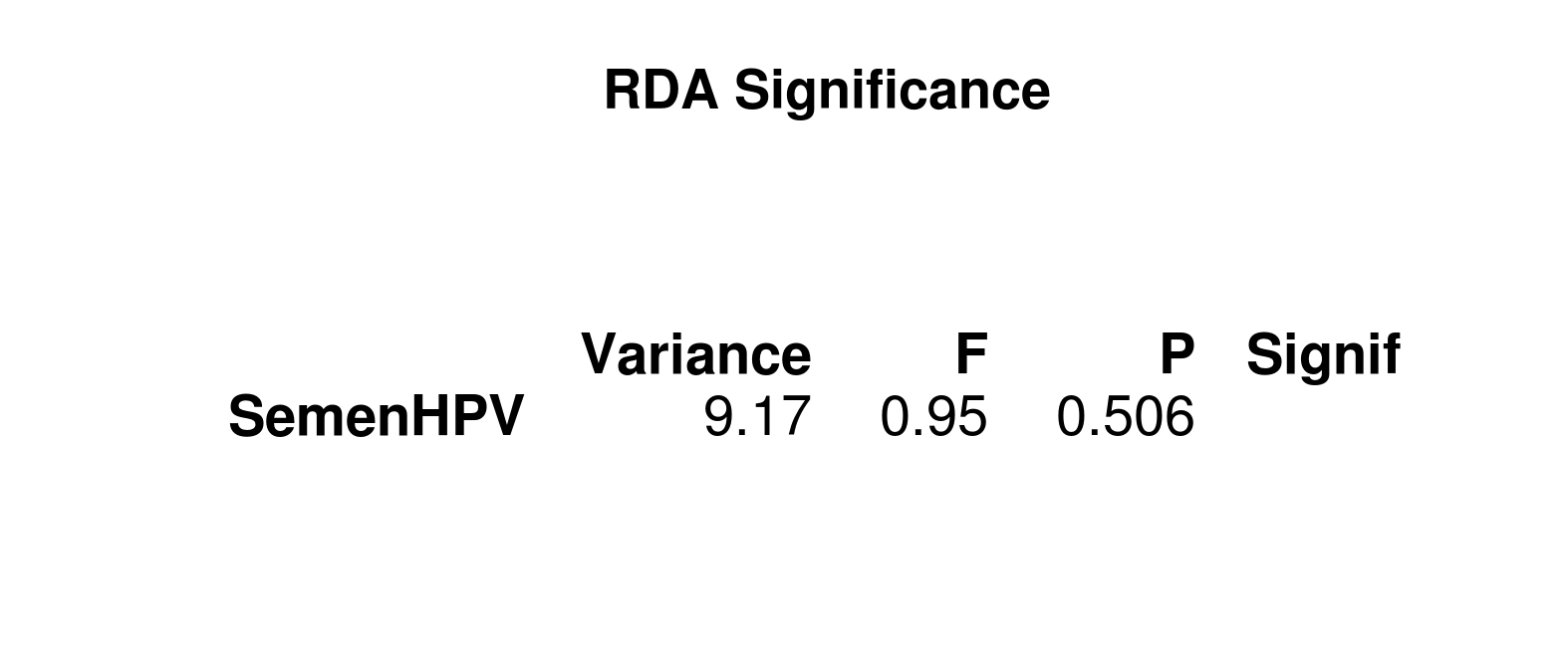

Supplement: Supplementary file 3 — Additional file 3: Supplementary Data 1. The beta-diversity estimations (0 = HPV-negative semen samples, 1 = HPV-positive semen samples). [file 12879_2021_6029_MOESM3_ESM.docx]
